# Ligands of Nanoparticles and Their Influence on the Morphologies of Nanoparticle-Based Films

**DOI:** 10.3390/nano14201685

**Published:** 2024-10-21

**Authors:** Jungwook Choi, Byung Hyo Kim

**Affiliations:** 1 Department of Materials Science and Engineering, Soongsil University, Seoul 06978, Republic of Korea; chchl678@soongsil.ac.kr; 2 Department of Green Chemistry and Materials Engineering, Soongsil University, Seoul 06978, Republic of Korea

**Keywords:** ligands, nanoparticles, nanoparticle-based film, morphology

## Abstract

Nanoparticle-based thin films are increasingly being used in various applications. One of the key factors that determines the properties and performances of these films is the type of ligands attached to the nanoparticle surfaces. While long-chain surfactants, such as oleic acid, are commonly employed to stabilize nanoparticles and ensure high monodispersity, these ligands often hinder charge transport due to their insulating nature. Although thermal annealing can remove the long-chain ligands, the removal process often introduces defects such as cracks and voids. In contrast, the use of short-chain organic or inorganic ligands can minimize interparticle distance, improving film conductivity, though challenges such as incomplete ligand exchange and residual barriers remain. Polymeric ligands, especially block copolymers, can also be employed to create films with tailored porosity. This review discusses the effects of various ligand types on the morphology and performance of nanoparticle-based films, highlighting the trade-offs between conductivity, structural integrity, and functionality.

## 1. Introduction

The rapid advancement of nanotechnology has significantly expanded the use of nanoparticles across various applications due to their unique physical and chemical properties [1,2]. These unique properties arise from the high surface area-to-volume ratio and small-sized nature of nanoparticles, where quantum mechanical effects dominate [3,4,5,6]. Because of these quantum effects and high surface area, semiconductor, metal, and magnetic nanoparticles below critical sizes exhibit phenomena such as quantum confinement effects, plasmonic resonance, and distinct magnetic behavior, respectively, which are not present in their bulk counterparts [7,8,9,10]. In recent several decades, breakthroughs in nanoparticle synthesis [11,12,13,14,15,16], surface functionalization [17,18,19,20,21,22,23], and characterization techniques [24,25,26,27] have enabled more precise control over their properties, allowing for the development of nanoparticles tailored for specific applications.

Attributed to the precise tunability of size and morphology of nanoparticles, which are independently synthesized before film formation, thin films from the nanoparticles offer several advantages over the films fabricated by conventional deposition methods such as physical vapor deposition (PVD) and chemical vapor deposition (CVD) [28]. First, since the synthetic methodologies of nanoparticles with diverse sizes, crystal structures, and compositions have been developed, thin films with demanded structures and compositions can be easily prepared from the nanoparticles [29]. Second, the regulation of surface structures of nanoparticles induced by using different passivating ligands allows controlling the energy state of interface atoms and charge transport efficiency of thin films [30]. Third, thin films from the surface-treated nanoparticles provide superior control over film morphology, allowing for fine-tuning of porosity, roughness, and surface area [31]. Moreover, the dopant distribution is easily controlled by ligand selection or postsynthetic doping, which is more difficult with PVD and CVD processes [32]. Nanoparticles can be integrated into thin films, with thicknesses ranging from tens of nanometers to several micrometers, using solution-phase deposition methods such as spin coating [33,34], dip coating [35,36], and spray coating [37,38,39]. The solution phase deposition methods follow deposition of nanoparticle dispersion, solvent evaporation, transport of nanoparticles, and self-assembly steps [40]. Winslow et al. revealed that the unbound ligands would affect the nanoparticle film growth as well as ligands bound to nanoparticles [41].

Nanoparticle-based films become central to the development of advanced electronics, including flexible electronics and wearable sensors [42,43,44], energy [45,46,47], and optical devices, including quantum dot displays [48,49]. For example, the indium-tin-oxide (ITO) nanoparticle-based transparent conducting oxide thin films show ultra-high mobilities, two times higher than those of conventional ITO films prepared by sputtering PVD methods [46]. This approach, fabrication of thin films from nanoparticles, offers a simple and cost-effective process for reconfigurable coatings, allowing optical properties to be adjusted and restored easily, improving versatility for nano-optical applications.

One of key factors influencing the properties of nanoparticle-based films is the ligands attached to the nanoparticle surfaces [50,51,52]. Surfactants with long hydrophobic chains are commonly used ligands for the synthesis of nanoparticles with high monodispersity [34,35,36]. The long-chain surfactant ligands are also essential for dispersing nanoparticles in hydrophobic solvents [53,54,55]. However, the surfactants act as a charge transport barrier because of the electrical insulating property of the long hydrocarbon chains [56]. Thus, charge transfer from a nanoparticle to an adjacent nanoparticle is interfered with by the long-chain ligands of the nanoparticles in the films.

Preparation of nanoparticle-based films with short particle-to-particle distance is critical for optimizing the performance of most nanoparticle-based devices. One promising approach to improving the performance of nanoparticle-based films is the careful modification of nanoparticle ligands before thin film deposition [1,50]. Ligand engineering enables researchers to control the morphology of films, which consequently affects their physical properties [57,58,59]. For example, films made from nanoparticles coated with long-chain organic surfactants tend to exhibit more voids and cracks, while those capped with short-chain ligands show fewer defects [60,61]. Ligand-exchange techniques have expanded the possibilities for tuning the morphology of nanoparticle-based films. Consequently, the electrical and optical performances of nanoparticle-based films can be optimized by tailoring the length and functional groups of the nanoparticle ligands. This strategy has proven effective in enhancing the performance of nanoparticle-based films across diverse applications such as transparent conducting oxide films [50,62,63], solar cells [64,65], and electrical devices [44,66,67]. It is worth noting that reducing cracks and voids is not universally beneficial. In specific applications, such as triboelectric sensor films, more porous films with high defect density allow for greater contact with sensing materials, improving the performance of the devices [68]. Thus, an optimizing balance between minimizing and controlling defects is required depending on the application.

This review aims to provide a comprehensive overview of the role of nanoparticle ligands in influencing the morphologies and properties of thin films fabricated using the nanoparticles. The novelty of this review lies in its detailed examination of how various ligand types affect the structural and functional properties of films for diverse applications. We first introduce various types of ligands and their functions in stabilizing nanoparticles that determine the uniformity of nanoparticle-based film. Subsequently, this review represents the morphological differences between thin films as a result of different ligand types. Afterward, we present representative examples showing the strong relationship between morphology and physical properties of the nanoparticle-based films, followed by future outlook and conclusions.

## 2. Types of Ligands

Ligands are molecules that stabilize the surface of nanoparticles, enhance dispersibility, and allow for precise control over the size, shape, and surface structure of the nanoparticles, thereby imparting additional functionalities [69,70,71]. The performances, properties, and stabilities of nanoparticles are governed by the ligands present on their surface [72,73,74]. In addition, ligands of nanoparticles are essential for obtaining monodisperse nanoparticles [75]. The ligands can act as barriers for monomer diffusion from the solution phase to the surface of nanoparticles during growth [76]. This function leads to a diffusion-controlled growth mechanism, which is a key mechanism in the formation of monodisperse nanoparticles [76,77,78].

**Figure 1:**
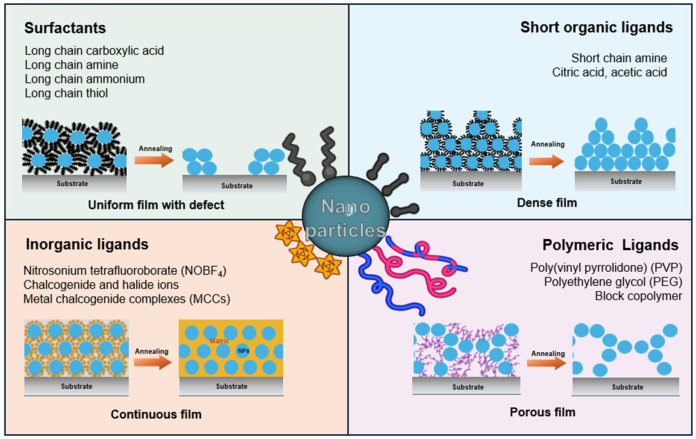
Schematics of types of ligands and morphology of the film influenced by ligands.

Ligands can be generally classified into two broad categories: organic and inorganic ligands (Figure 1). Organic ligands consist of surfactants with long carbon chains and hydrophilic functional groups such as -COOH, -NH_2_, -SH, and -OH [45,70]. The appropriate selection of organic ligands allows obtaining monodisperse nanoparticles by preventing agglomeration and achieving diffusion-controlled growth mechanisms. The shapes of nanoparticles have also been controlled by using specific ligands that can direct growth along certain crystallographic directions resulting from their different affinities for exposed facets [43,74].

Inorganic ligands, typically comprising metal or semiconductor compounds, include S^−^, CO_2_^−^, BF_4_^−^, I^−^, Cl^−^, NO_2_^−^, and metal chalcogenide complexes [48,79]. These ligands are essentially useful for fabricating all inorganic nanoparticle-based films, where their superior electrical conductivity compared to organic ligands is advantageous [80,81]. The type and nature of ligands directly affect the final properties of nanoparticles, underscoring the importance of careful ligand selection in the fabrication of devices using nanoparticle-based thin films (Figure 1).

We briefly present another classification of ligands based on the ligand coordination mechanism: L-type, X-type, and Z-type ligands. L-type ligands, including amine and phosphine, are neutral electron donor molecules that coordinate to the nanoparticles surface through a lone pair of electrons, typically forming dative bonds with metal atoms. X-type ligands have anionic functional groups that bind to cationic surface atoms of nanoparticles along with charge compensation. Carboxylates, phosphonates, halides, and thiolates are representative X-type ligands. Z-type ligands are identified as metal-ligand complexes that also bind to the nanoparticle surface. Although the coordination chemistry-based classification of ligands is widely used, we will focus on classifying ligands based on their length and composition in this review. This is because the length and composition of ligands play a critical role in determining the morphology of nanoparticle-based thin films. Detailed information on the L-type, X-type, and Z-type ligands can be found in previous studies and reviews [82,83,84,85].

### 2.1. Surfactants

Surfactants, which are molecules with both hydrophilic functional groups and hydrophobic long hydrocarbon chains, are commonly used in the synthetic methods of nanoparticles in hydrophobic media such as thermal decomposition [86,87,88,89], solvothermal method [90,91], and non-hydrolytic sol-gel reaction [92]. The co-existence of both hydrophobic and hydrophilic parts in a molecule allows the surfactants to act as stabilizers of nanoparticles [93]. The hydrophilic groups of the surfactants bind to the surface of nanoparticles with high polarity, especially in metal oxide nanoparticles [94], through a coordination bond or ionic bond, whereas the hydrophobic groups of the surfactants allow the surfactant-capped nanoparticles to be dispersed in hydrophobic solvents [94,95]. Common hydrophilic functional groups in surfactants for nanoparticles include carboxylic acid (-COOH), amine (-NH_2_), thiol (-SH), hydroxyl (-OH), and cationic ammonium groups (-NH_3_^+^) [50,70,96].

Surfactants containing carboxylic acid groups, such as oleic acid, are extensively used in the synthesis of metal oxide nanoparticles [97,98,99]. It is known that the bonding between the surfactants and metal surfaces is explained as a coordination bond by coordinating unpaired electrons of oxygen anions of carboxylate groups to the metal cations on the surface of nanoparticles after deprotonation of the carboxylic acid groups of the surfactants [100,101]. The oxygen anions of the carboxylate groups are regarded as hard bases. On the basis of hard-soft-acid-base theory, the hard-base nature of carboxylate allows them to coordinate effectively with hard acids such as metal cations with high oxidation numbers and small ionic sizes [102]. In this regard, synthesis of 1st row transition metal oxide nanoparticles with high oxidation numbers requires surfactants with carboxylic acid [98,103]. Surfactants with carboxylic acid groups are commonly used for synthesizing transition metal oxide nanoparticles like TiO_2_, Fe_3_O_4_, and Mn_2_O_3_ [104,105,106,107,108].

**Figure 2:**
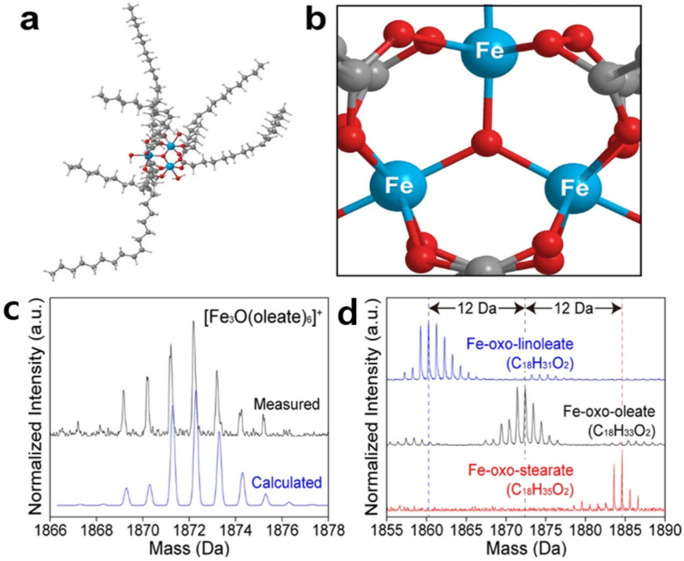
(**a**) Computed trinuclear-oxo carboxylate structure of iron-oleate complex and (**b**) core of the iron-oleate complex. (**c**) Isotope calculation of the main peak at *m*/*z* = 1872, verifying the structure. (**d**) MALDI-TOF mass spectra of iron-oxo carboxylates having different numbers of double bonds in the ligand. (reproduced with permission [115], Copyright American Chemical Society).

Oleic acid is the most widely used surfactant for the solvothermal and hydrothermal synthesis of metal oxide nanoparticles. The long-chain hydrocarbon of oleic acid increases particle-to-particle distances and imparts strong hydrophobicity to the oleic acid-capped nanoparticles, resulting in high dispersity of the nanoparticles in hydrophobic solvent. In addition, the bend structure of oleic acid, originated from the cis-alkene double bond in the 9th carbon-carbon bond, weakens the inter-packing between long hydrocarbon chains, reducing van der Waals interaction between surfactant molecules and improving the dispersity of nanoparticles [109]. The synthesis of nanoparticles in the presence of oleic acid often led to a narrow particle size distribution compared to that of nanoparticles synthesized without a surfactant. The narrow particle size distribution of the nanoparticles capped by oleic acid is explained by a diffusion-limited growth mechanism, which is attributed to the retardation of monomer diffusion to the surface of nanoparticles through long hydrocarbon chains of oleic acid with high hydrophobicity [110,111,112]. A number of papers have been published for the synthesis of monodisperse metal oxide nanoparticles using oleic acid as a surfactant [113]. Several studies show that oleate not only acts as a surfactant but also involves in reaction mechanisms by stabilizing metal ion precursors for the synthesis of monodisperse nanoparticles. Hyeon and coworkers assumed that the iron-oleate complexes are formed before the nucleation of iron oxide nanoparticles using Fe(CO)_5_ precursors and oleic acid ligands [114]. The assumption is verified by mass spectrometric analysis [115]. Matrix-assisted laser adsorption/desorption-time of flight (MALDI-TOF) mass spectrometry shows that mass spectra for iron-oleate complexes appear consistently before the nucleation of iron oxide nanoparticles from different precursors, including iron chloride, iron acetylacetonate, and iron nitrate (Figure 2). The transition metal-oleate complexes can be easily synthesized by a simple reaction between metal chloride and sodium oleate. Iron-oleate complexes have proven to have a trinuclear-oxo carboxylate structure (Figure 2) [115]. Due to the high stability and low price of the metal-oxide complexes, large-scale synthesis of highly monodisperse oleate-capped metal oxide nanoparticles can be synthesized by thermal decomposition of the complexes [116,117,118,119,120]. 

Surfactants having amine groups, such as oleylamine, are also frequently used to produce metal, metal oxide, and metal chalcogenide nanoparticles [121]. Oleylamine has a long and bent hydrophobic hydrocarbon chain as well as oleic acid. Long-chain amine ligands often involve nucleation and growth mechanisms of nanoparticles via the formation of metal-amine complexes [122], reduction of precursors at high temperatures [123], and aminolysis reactions [124].

The amine group of oleylamine is on the borderline between a hard base and a soft base. Thus, the binding affinity of oleylamine to the surface of metal oxide, which exhibits properties of Lewis hard acid, is weaker than that of oleic acid. Due to the moderate binding affinities, oleylamine is usually used with other co-surfactants, such as oleic acid. For example, Fe_3_O_4_ nanoparticles have been synthesized using Fe(acac)__3__ as a precursor in the presence of 1,2-dodecanediol, oleylamine, oleic acid, and dibenzyl ether [125]. The combination of oleylamine and oleic acid surfactants forms a protective layer around the particles, effectively preventing their aggregation. As the amine group is on the borderline of hard and soft bases, the long-chain amine surfactants are also used to passivate metal and semiconductor nanoparticles that exhibit soft acid properties. For instance, coating palladium nanoparticles with hexadecylamine enhances their dispersion and stability [126]. However, the amine group shows weaker affinity to the surface of soft, acidic noble metal nanoparticles than that of thiols, a softer base, resulting in amine-modified nanoparticles being larger than thiol-functionalized nanoparticles.

The combination of both oleylamine and oleic acid is often used to regulate the shape of nanoparticles [127]. Due to the appropriate binding affinity of oleylamine to the nanoparticle surface, the surface energy of the nanoparticles can be controlled by varying the ratio of oleylamine and oleic acid. By utilizing surface energy engineering, non-spherical nanoparticles can be synthesized by inducing each facet of nanoparticles to have a different growth rate [128]. For instance, rod and spherical-shaped TiO_2_ nanoparticles were synthesized from titanium isopropoxide precursors using oleic acid and oleylamine as co-surfactants [129]. Similarly, Mn-doped ZnO nanoparticles with hexagonal structures were synthesized by thermal decomposition of zinc acetate and manganese acetate precursors in the presence of oleylamine and oleic acid. The ratio of oleylamine to oleic acid is crucial in controlling the particle morphology. By adjusting the ratio, Mn-doped ZnO nanoparticles with various morphologies, hexagonal nanopyramids, nanodisks, and irregular nanospheres were successfully obtained, highlighting the significant role of oleylamine in promoting particle growth and shape modification [130].

Cationic long-chain ammonium surfactants, cetyltrimethylammonium bromide (CTAB), are often employed as shape-directing agents, similar to long-chain amine. CTAB has been widely used in the synthesis of both spherical and anisotropic nanoparticles, especially noble metal nanorods [131]. Murphy and coworkers produced gold nanorods with controlled aspect ratio and size using a seed-mediated wet chemistry approach using CTAB molecules. In the nanorod synthesis, CTAB forms micellar templates for the anisotropic growth of nanostructures, encouraging the formation of rod-like particles [132]. CTAB preferentially binds to the (100) crystal planes of the nanorods, promoting anisotropic crystal growth along the (111) facets with lower ligand coverage [133]. The strategy of using a surfactant mixture, such as combining sodium oleate with CTAB, proved to be effective in producing gold nanorods with tunable dimensions [134]. Other long-chain ammonium halide surfactants have also been used to control the morphology of gold nanorods. The use of etyltrimethylammonium chloride (CTAC) and decyltrimethylammonium bromide (DTAB) in the synthesis of gold nanomaterials produces nanorods with short aspect ratios and lower yields [135]. CTAC molecules have also been utilized in the synthesis of triangular gold nanoplatelets in combination with iodine ions from potassium iodide [136]. The growth of these nanoplatelets is driven by the preferential binding of I− ions to the Au(111) facet, as well as through oxidative etching, which removes less stable nuclei. Tetradecyltrimethylammonium bromide (TTAB) forms micelles and has been used to synthesize platinum nanoparticles with cubic and cuboctahedral shapes during a borohydride reduction [137]. The alkyl ammonium halides weakly bind to the nanoparticle surface, enabling easy ligand exchange with more strongly binding ligands.

Ligands containing thiol groups play an important role in the synthesis of noble metal nanoparticles due to their strong affinity for metal surfaces [138]. According to the hard-soft-acid-base theory, the thiol group, a soft base, binds strongly to soft acids, such as zero-valent metal atoms with large polarizability. In this regard, thiol groups strongly bind to the surface of noble metals such as gold, silver, and platinum. Due to the strong interaction between gold and thiol, crystal growth onto the surface of gold nanoparticles is hindered by the coordinated thiol groups, resulting in small-sized gold nanoparticles, less than 5 nm [139,140,141].

Phosphine-passivated metal nanoparticles are synthesized by reducing metal precursors in the presence of phosphine ligands such as triphenylphosphine. The phosphine acts as both a precursor and a reductant by oxidizing to phosphine oxide [142]. The phosphine ligands form weak interactions with metal surfaces, leading to low nanoparticle stability [143]. This instability makes phosphine ligands easy to exchange with other ligands that bind more strongly to the metal. Unlike ligand exchange of thiol-passivated gold nanoparticles, phosphine ligands are fully replaced with other kinds of ligands with simple reactions [144]. The exchange of phosphines to thiols significantly enhances the stability of the nanoparticles [145]. In contrast, exchanging phosphines with amines produces less stable gold particles, but an interesting aspect of this process is that the particles grow in size during the modification [143]. The stability issue associated with phosphine ligands can be addressed by using polyphosphine ligands. For example, palladium nanoparticles have been effectively stabilized with di- or triphosphine [146]. These multiphosphine ligands have been shown to strongly coordinate with the surface of palladium particles.

### 2.2. Short Organic Ligands

The use of short organic ligands has been explored to improve the electrical and catalytic properties of nanoparticles [50,51,73]. These ligands, characterized by shorter carbon chains, increase the packing density of nanoparticle-based films by reducing particle-to-particle distance. Metal nanoparticles have been modified with short-chain amines and ammonium. Ammonium ions with short chain lengths of 4–8 carbons have been used to stabilize transition metal nanoparticles [147,148,149]. In electrochemical synthesis, these ammonium surfactants act as both electrolytes and stabilizers, producing size-controlled and monodisperse nanoparticles in the range of 1.2 to 5 nm [150,151,152]. Short-chain organic ligands, such as acetic acid, were used to stabilize nanoparticles [153].

However, the decreased particle-to-particle distance of nanoparticles capped with short organic ligands exhibits high van der Waals interaction between nanoparticles, often causing particle aggregation [70,154]. Such aggregation, occurring after the replacement of long-chain ligands with short-chain ones, can influence surface tension and alter the structural properties of the nanoparticle films [155]. Therefore, preventing aggregation while maintaining the dispersity of nanoparticles is crucial when adopting short-chain ligands. Selection of short-chain ligands that provide sufficient repulsive forces between nanoparticles is necessary to ensure stability.

One effective strategy for stabilizing nanoparticles with short organic ligands is to utilize electrostatic repulsion [70]. In this approach, charged ligands are introduced to nanoparticle surfaces, creating electrostatic repulsive forces that prevent close contact between nanoparticles, thereby inhibiting aggregation [156]. This strategy is particularly beneficial for stabilizing nanoparticles in polar solvents, where the combination of short ligands and electrostatic repulsion ensures colloidal stability [79]. Citrate ions, for instance, with three carboxylate groups, are commonly used in the synthesis of metal nanoparticles. The citrate-capped nanoparticles exhibit negative zeta potential due to the anionic carboxylate groups of the charged ligands [157]. The electrostatic repulsion from the negative surface charge of nanoparticles prevents aggregation, ensuring the nanoparticles are dispersed well in aqueous media [158].

The hydrophobic nanoparticles capped with long-chain surfactants can be transferred to hydrophilic media via replacement of the surfactants with short organic ligands. The ligand exchange is conducted by reacting the surfactant-capped nanoparticles with an excess amount of short organic molecules. For example, oleic acid or oleylamine-capped nanoparticles can be dispersed in an aqueous solution by exchanging the long-chain ligands for hydrophilic ligands such as citrate [52,125]. The phase transfer is essential for applications requiring aqueous systems, including biomedical imaging [159].

The replacement of ligands is confirmed by analyzing the chemical structure of ligands using Fourier transformed-infrared (FT-IR) and nuclear magnetic resonance (NMR) spectroscopy. For examples, oleic acid-coated iron oxide nanoparticles show FT-IR peaks of 2920 cm^−^^1^ and 2850 cm^−^^1^ for alkyl C-H stretch, 1707 cm^−^^1^ for carboxylic C=O stretch, 1450 cm^−^^1^ and 1348 cm^−^^1^ for C-O stretch, which indicated bound asymmetric O-C-O and symmetric [160]. However, after modification with shorter ligands such as (3-aminopropyl) triethoxysilane (APTES), taurine, and 2-mercaptoethanol, these peaks diminish or disappear, suggesting ligand exchange. New peaks, such as APTES at 1520–1540 cm^−^^1^ for taurine and 956–1110 cm^−^^1^ for sulfur-containing ligands, reflect binding of functional groups to the nanoparticle surface. Peak shifts and splitting indicate interactions like sulfur-oxygen or phosphonate bonding. These spectral changes qualitatively confirm the exchange of long ligands with shorter ones [160]. NMR has also been used for the confirmation of the successful ligand exchange. For gold nanoparticles, the citrate primary ligands exhibit an NMR peak around 2.6 ppm (J = 16 Hz). After the replacement of the ligands with other carboxylic acids, new NMR peaks appeared at 1.91 ppm for acetate-capped nanoparticles and 2.27 and 3.44 ppm for acetoacetate-capped nanoparticles [161]. 2-dimensional NMR, especially diffusion ordered spectroscopy (DOSY), has also been used to determine the bonding between nanoparticles and ligands [162].

### 2.3. Inorganic Ligands

Nanoparticles capped by inorganic ligands are often prepared via the exchange of bulky organic ligands, which are typically introduced during the nanoparticle formation, with inorganic ligands. The ligand exchange offers significant advantages, primarily in improving conductivity and reducing surface trap sites [52,79]. The nanoparticles with inorganic ligands exhibit high conductivity due to efficient electron transfer between nanoparticles [52]. Inorganic ligands are inherently more conductive than organic ligands, allowing for fast electron transport. Moreover, due to the small-sized nature of inorganic ligands, electrons of a nanoparticle are readily transported to an adjacent nanoparticle through the reduced particle-to-particle distance [50,52,55]. When nanoparticles capped by inorganic ligands are deposited, densely packed nanoparticle-based films can be obtained [45,64,163]. The shorter interparticle distance and reduced tunneling barrier between nanoparticles with inorganic ligands result in higher electron mobility, improving the overall performance of nanoparticle-based films in electronic devices [55,66]. Additionally, the wide variety of available inorganic ligands allows for fine tuning of the physicochemical properties of nanoparticles, enabling preparation of nanoparticle-based films with tailored functionalities.

**Figure 3:**
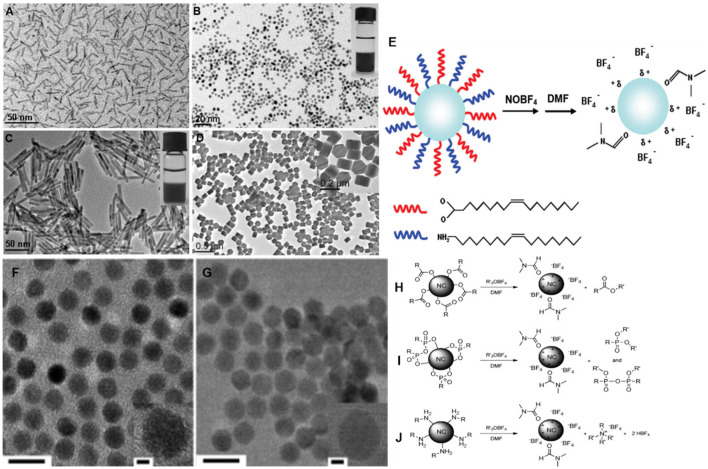
TEM images of the BF_4_^−^-treated (**A**) TiO_2_ nanorods, (**B**) FePt nanopaticles, (**C**) Bi_2_S_3_ nanorods, and (**D**) NaYF_4_ nanoplates dispersed in DMF. (**E**) Schematics of the ligand exchange process using NOBF_4_ (reproduced with permission [164], Copyright American Chemical Society). (**F**,**G**) TEM images of Et_3_OBF_4_-treated PbSe nanoparticles dispersed in acetonitrile. (**H**) Schematics of the ligand stripping of (**H**) carboxylate-, (**I**) phosphonate-, and (**J**) amine-capped nanocrystals with Et_3_OBF_4_. (reproduced with permission [165], Copyright Wiley-VCH).

Nitrosonium tetrafluoroborate (NOBF_4_) is often used to replace bulky organic ligands to stabilize nanoparticles in polar solvents like N,N-dimethylformamide (DMF), dimethylsulfoxide (DMSO), or acetonitrile [164]. NOBF_4_ is an ionic compound composed of BF4^−^ and NO+. It is known that BF4^−^ acts as a stabilizing ligand, although it has a relatively weak binding affinity to metal oxide surfaces. NO^+^ serves as a good leaving group during the ligand exchange process, and it is easily reduced to NO and NO_2_, which facilitates the attachment of BF_4_^−^ to the nanoparticles. Additionally, through the combination of BF_4_^−^ and the co-solvent such as DMF, hydrophilicity can be induced to the nanoparticle surface, leading to the stabilization of nanoparticles (Figure 3). Ligand stripping with NOBF_4_ is important for the nanoparticles to be used in catalysts because NOBF_4_ effectively removes the long hydrocarbon ligands that cover the nanoparticle surface, which typically interfere access of molecules to the active sites in catalysts. This process dramatically enhances the active surface area of platinum by exposing uncoordinated platinum atoms. Thus, the use of NOBF_4_ as a ligand in platinum alloy nanoparticles, such as FePt and CoPt3, has a significant improvement on their catalytic performance. Unlike conventional ligand-removing methods like UV/ozone treatment or thermal annealing, which are either limited to thin films or risk agglomeration, ligand exchange with NOBF_4_ preserves the morphology and solution processability of the nanoparticles while efficiently activating their catalytic sites. The advantages also make the NOBF_4_-treated nanoparticles highly suitable for electrocatalytic applications, as demonstrated by the improved hydrogen accessibility and increased surface activity in both FePt and CoPt_3_ nanoparticles [164].

Despite the versatility of NOBF_4_ as short inorganic ligands for various oxide nanoparticles, the high oxidative reactivity and Lewis acidity of NO^+^ make NOBF_4_ unsuitable for oxidation-prone chalcogenides nanoparticles. Milliron and coworkers introduced Meerwein’s salt, composed of trialkyl oxonium ions (Et_3_O^+^) paired with BF_4_^−^ anions (Figure 3). The Meerwein’s salt effectively strips ligands from nanoparticle surfaces through alkylation, replacing them with anions without oxidative damage. Thus, Meerwein’s salt can be used as a short inorganic ligand for chalcogenide nanoparticles such as CdSe and PbSe [165].

In some cases, inorganic ligands with the same constituent elements as the nanoparticles are used for the fabrication of nanoparticle-based films to obtain nanoparticle-based films without impurities [66]. When ligands are not fully removed during the film fabrication process, they can act as impurities in the nanoparticle-based films, affecting the properties of the films. By using ligands made of the same atoms as the nanoparticles themselves, impurity-free nanoparticle-based films can be achieved [166]. Sulfide (S^2^^−^) is commonly used as inorganic ligands for metal sulfide semiconductor nanoparticles [167]. Because sulfur is a constituent element of metal sulfide semiconductors, S^2−^-passivated metal sulfide nanoparticles show low surface traps that decrease the number of exciton by recombination [167,168]. In addition, the nanoparticle-based films from S^2−^- functionalized metal sulfide nanoparticles show extremely high performances because the S^2−^ ligands do not act as impurities. CdSe/CdS core/shell quantum dots (QDs) are successfully transferred into an aqueous medium by ligand exchange with S^2^^−^ ligands [169]. The QDs are well dispersed in aqueous solution with electrostatic repulsion between S^2^^−^-capped QDs [169].

Halide ions, such as chloride (Cl^−^), bromide (Br^−^), and iodide (I^−^), are used as ligands to stabilize nanoparticles [170]. These anions can bind to the surface of metal nanoparticles, passivating the surface and reducing surface energy, which helps prevent uncontrolled growth and aggregation [171]. Halide ligands are particularly useful for controlling the morphology and size distribution of nanoparticles during synthesis. In addition, the halide ions serve as effective passivating agents, where surface defects need to be minimized [172]. By binding to unsaturated metal atoms on the nanoparticle surface, halides can neutralize surface charge and prevent the formation of trap states, which is crucial for applications requiring high electronic performance, such as in optoelectronic devices. Halide ions can also influence the shape and structure of nanoparticles. During the growth of metal nanoparticles, halides can preferentially adsorb on specific crystallographic planes, leading to anisotropic growth and formation of nonspherical nanoparticles, such as nanorods, nanowires, or nanoplates, depending on the specific halide and synthesis conditions. [172].

Metal chalcogenide complexes (MCCs) are another class of inorganic ligands that play a vital role in stabilizing and functionalizing colloidal nanoparticles. Typical MCC ligands include chalcogenidometallate molecules such as Sn_2_S_6_^4^^−^, In_2_Se_4_^2^^−^, and AsS_3_^3^^−^, with common counterions like Na^+^, K^+^, NH_4_^+^, and N_2_H_5_^+^, offering structural complexity. MCCs are particularly valuable due to their ability to significantly reduce inter-nanoparticle spacing, thereby enhancing electronic coupling between adjacent nanoparticles. For instance, replacing long-chain organic ligands like dodecanethiol on gold nanoparticles with Sn_2_S_6_^4^^−^, increases conductivity up to 200 S/cm, which is 11 times higher than that of dodecanethiol-capped nanoparticles. This increased conductivity results from strong electronic coupling facilitated by the close packing of Sn_2_S_6_^4^^−^-capped nanoparticles. The electronic properties of MCC-capped nanoparticles are further evidenced by UV-visible absorption spectroscopy. While UV-vis spectra of dodecanethiol-capped gold nanoparticles in both solution and nanoparticle-based films show localized plasmon resonance peaks, that of MCC-capped nanoparticles reveals that they are delocalized. This delocalization is indicative of strong electronic coupling among the nanoparticles. Similar effects are observed in quantum-confined semiconductor nanoparticles, such as CdSe, where MCC capping results in a red-shift and broadening of the excitonic features in thin films, indicating wavefunction overlap between closely packed nanoparticles [79].

Polyoxometalates (POMs), large metal-oxygen anionic clusters, are another versatile class of inorganic ligands for stabilization and functionalization of nanoparticles. POMs stabilize nanoparticles in aqueous solutions due to their high charge density, which provides robust electrostatic stabilization mechanisms. This stability is crucial for maintaining the dispersion and preventing the aggregation of nanoparticles in solution, especially under varying environmental conditions. POMs are also recognized for their multifunctional capabilities. They can act as electron acceptors, enhancing the photocatalytic and electro-catalytic activities of nanoparticles. This is particularly advantageous in applications like photocatalytic water splitting or electrocatalysis, where efficient charge transfer and high catalytic activity are required. POMs have been shown to improve the photocatalytic performance of Fe_2_O_3_ and TiO_2_ nanoparticles by serving as sinks for photogenerated electrons, thus improving the overall efficiency of the photocatalytic process. Additionally, POMs have the ability to form new inorganic phases after thermal annealing or chemical treatment [173]. Hybrid-type nanoparticle-based films with novel properties can be fabricated by using the POM-passivated nanoparticles, which can further apply to dual-band electrochromic devices [174].

### 2.4. Polymeric Ligands

Polymers can stabilize nanoparticles by engaging in both steric and electrostatic interactions, attaching to the nanoparticle surface through either physisorption or chemisorption. During the synthesis of nanoparticles, polymers may preferentially bind to specific crystallographic planes on the surface, thereby influencing anisotropic crystal growth or serving as a matrix for the formation of nanoparticles. The preferential binding can be adjusted by applying block copolymers that consist of different polymeric chains with different binding affinity to the nanoparticle surfaces [50].

Poly(vinyl pyrrolidone) (PVP)-stabilized gold nanoparticles display altered optical properties due to energy transfer between PVP and the gold nanoparticle core. When dispersed in hot water, PVP functions as both a surface ligand and a facilitator for the clustering and growth of polygonal gold nanoparticles, utilizing small polymer templates. PVP is also a key component in the polyol reduction method with ethylene glycol for synthesizing cuboctahedral palladium nanoparticles (4–8 nm). The literature provides an extensive review of the roles of PVP in colloidal nanoparticle synthesis. Similarly, poly(vinyl alcohol) (PVA) has proven to be an effective ligand in nanoparticle synthesis, such as in the formation of copper nanoparticles via citrate reduction in the presence of sodium formaldehyde sulfoxylate and PVA [175].

Polyethylene glycol (PEG) has been employed to stabilize copper nanoparticles during reduction with borohydride/ascorbic acid, which also induces shifts in the plasmon band to around 560–570 nm. In aqueous solutions containing alkyl thioether end-functionalized poly(methacrylic acid), near-monodisperse gold nanoparticles (1.4–4 nm) were synthesized. Additionally, polyelectrolyte-protected gold nanoparticles of various sizes have been synthesized directly by heating AuCl_4_– in an aqueous solution containing amine-based polyelectrolytes such as poly(ethylenimine) and poly(allylamine hydrochloride) [175].

In the presence of a triblock copolymer Pluronic P123 (PEO19–PPO69–PEO19), reducing palladium salts yields spherical palladium nanoparticles. The Pluronic P123 block copolymer effectively stabilizes the palladium nanoparticles and prevents aggregation. The poly(ethylene oxide) (PEO) segment of the triblock copolymer ligands interacts with metal ions. In the reduction process, the PEO part of the Pluronic copolymer acts as a reducing agent for metal ions. However, in low-pH conditions, the presence of H+ ions disrupts the interaction between PEO and nanoparticle surface. This disruption reduces the number of nucleation sites, leading to fewer nuclei and consequently larger nanoparticles. Additionally, the decreased coordination ability of the Pluronic copolymer at low pH further limits its capacity to stabilize growing nanoparticles, which can result in the formation of dendritic structures under more acidic conditions. The resulting palladium nanoparticles were highly crystalline, with a spherical morphology and an average diameter of approximately 4.8 nm. Additionally, the nanoparticles remained well-dispersed in water, forming a stable colloidal suspension that showed no signs of precipitation even after five months at room temperature. This method demonstrates the effectiveness of using block copolymers like Pluronic P123 as ligands in nanoparticle synthesis, offering excellent control over particle size, stability, and crystallinity [176].

## 3. Morphology Change of Nanoparticle-Based Films with Different Ligands

Thin films fabricated from dispersed nanoparticles are influenced by the length and composition of ligands. The dispersity of nanoparticles, which depends on both the structure of ligands and the size of the nanoparticle core, is also an important factor in determining the final morphologies of nanoparticle-based films. Morphological differences in the films usually lead to differences in their properties. Sometimes, new properties emerge or are enhanced when nanoparticles are deposited as a film. For example, close packing of gold nanoparticles in a film increases localized surface plasmon resonances by reducing the distance between particles [177,178,179]. These results demonstrate that the geometry of the film affects the physical properties of nanoparticles within the film.

### 3.1. Morphologies of Films from Surfactants-Passivated Nanoparticles

The uniformity and roughness of a nanoparticle-based film are dependent on the dispersity of nanoparticles as well as the deposition conditions during the film fabrication. The nanoparticle-based films made from nanoparticles passivated with surfactants such as oleic acid and oleylamine exhibit high uniformity with low roughness because of the high dispersity of the surfactant-capped nanoparticles. The long-chain surfactant ligands prevent aggregation by coating the surface of nanoparticles and enhance particle dispersion. The high dispersity of the surfactant-passivated nanoparticles allows for the formation of uniform films with low roughness through solution-based deposition methods such as spin coating, dip coating, blade coating, and roll-to-roll deposition. Films made from oleic acid-capped PbS nanoparticles had smooth and uniform surfaces because of the high dispersity of the nanoparticles. The surfactants control the interparticle distance by modulating the interaction between nanoparticles, which is influenced by the ratio of oleic acid and oleylamine used [180].

However, the presence of long surfactants reduces the conductivities of resulting nanoparticle films as the surfactants act as insulating layers that impede charge transfer. The thick coating around each nanoparticle created by long surfactants leads to more spaced-out arrangements within the film. This large separation between nanoparticles reduces electronic coupling and negatively affects both the conductivity and stability of the film. Long surfactants, therefore, are required to be treated to control the spatial arrangement while preventing the aggregation of nanoparticles [181]. De Roo et al. studied the effect of nanoparticle ligand length on the morphology of hafnium oxide nanoparticle-based films and their electrical properties for memristor applications [182]. The authors prepared hafnium oxide nanoparticles passivated with phosphonic acid of different chain lengths, including oleyl-, n-octadecyl-, and 2-ethylhexyl-phosphonic acid, and deposited memristor films using these nanoparticles. Among the films, that from nanoparticles with 2-ethylhexyl phosphonic acid, which has a relatively short chain, showed the best performances, achieving a record low set voltage of 1.0 ± 0.3 V. In contrast, longer ligands such as oleyl and n-octadecyl phosphonic acid required higher set voltages around 4.4 V. The results indicate that the regulation of ligand length is critical for fabricating promising memristors for flexible electronic applications. The colloidal stability of the hafnium oxide nanoparticles is also affected by ligand length because of the trade-off between ligand length and stability. While 2-ethylhexyl phosphonic acid provides both low voltage operation and colloidal stability for hafnium oxide nanoparticles (3.75 nm in diameter), larger nanoparticles (5 nm) require slightly longer ligands, like 2-hexyldecyl phosphonic acid, to maintain dispersion stability. Thus, selecting an appropriate ligand length is important to balance colloidal stability and electrical conductivity simultaneously [182].

The long-chain insulating ligands can be removed by thermal annealing [183,184], in-film ligand stripping [185,186], and plasma treatment [187]. However, the ligand removal in film state often causes defects in nanoparticle-based films, such as cracks and voids, during the film fabrication process [188,189]. These defects negatively impact the performance of devices consisting of nanoparticle-based films by degrading the mechanical, electrical, and optical properties of the films [190,191,192]. For example, in photovoltaic devices, defects in the nanoparticle-based films can act as recombination centers for electrons, significantly lowering power conversion efficiency [193,194]. Similarly, for optical devices, defects with the sizes of hundreds of nanometers can cause light scattering, which reduces transparency and optical performances [48,195]. In electronic devices, cracks within nanoparticle-based films can disrupt charge transport pathways, leading to reduced electrical conductivity and device efficiency [196,197].

**Figure 4:**
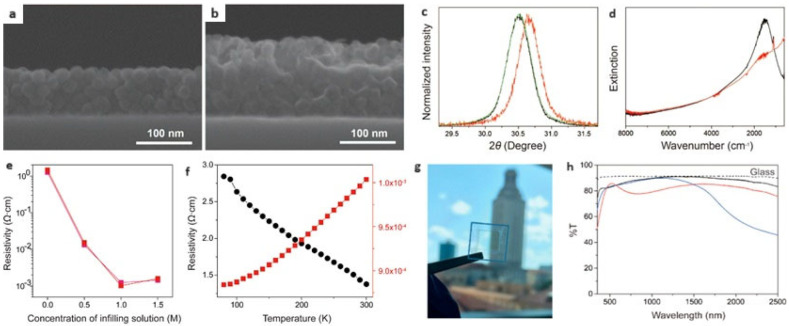
(**a**) cross-sectional scanning eletron microscopy (SEM) image of Ce:In_2_O_3_ nanoparticles film after thermal annealing, which shows cracks and voids in the film. (**b**) SEM image of a composite film after in-filling of 1 M indium oxide and thermal annealing. (**c**) (222) peak of XRD patterns of (green) 21 nm-sized Ce:In_2_O_3_ nanoparticles, (black) annealed Ce:In_2_O_3_ nanoparticle film, and (red) a composite film after in-filling. (**d**) Fourier transformed infrared (FTIR) spectra of (black) Ce:In_2_O_3_ nanoparticle film and (red) composite film after in-filling. The FTIR spectra of the composite film indicate electron delocalization. (**e**) Resistivity of nanoparticle film and composite films prepared by in-filling 0.5, 1, and 1.5 M of indium combustion solution onto the NC arrays. (**f**) Temperature-dependent resistivity data of (black) a Ce:In_2_O_3_ nanoparticle film and (red) a composite film after in-filling. (**g**) Highly transparent Ce:In_2_O_3_ NC-based composite film. (**h**) Transmission spectra of (black) a Ce:In_2_O_3_ nanoparticle film, (red) an in-filled composite film, (blue) a Sn:In_2_O_3_ nanoparticle-based composite film, and (black dot) a glass substrate. (Reproduced with permission [46], Copyright American Chemical Society).

The low charge carrier mobility of the thermally annealed nanoparticle-based films can be improved by filling these gaps with a precursor solution and subsequent annealing. The in-filling process created a continuous, conductive matrix that minimized electron trapping at surface defects and enhanced electron delocalization. This improved charge mobility and transformed the transport behavior of the nanoparticle-based films from hopping mechanisms to metal-like transport, as evidenced by a positive temperature coefficient of resistance. Figure 4 shows the morphology and physical properties of nanoparticle-based composite films by in-filling indium oxide matrix with Ce:In_2_O_3_ nanoparticle arrays [46]. While the nanoparticle-based films without infilling exhibited low electron mobility due to the localized electrons, the composite films showed low resistivity and high electron mobility of ~58 cm^2^/Vs, which is two times higher than the commercial indium-tin oxide films. The nanoparticle-based composite films were used to transmit conducting oxide for smart window applications.

### 3.2. Morphologies of Films from Nanoparticles Passivated with Short Organic Ligands

The morphology of films fabricated from nanoparticles passivated with short organic ligands exhibits a trend opposite to those from nanoparticles passivated with long chain ligands. The nanoparticle-based films from short-chain ligand-capped nanoparticles display a reduced particle-to-particle distance, increasing the conductivity of the nanoparticle-based films. For instance, the conductivity of nanoparticle-based Sn:In_2_O_3_ films was improved by exchanging long-chain ligands (oleate and oleylamine) with short-chain molecules, tetrabutylammonium hydroxide (TBAOH) [63].

**Figure 5:**
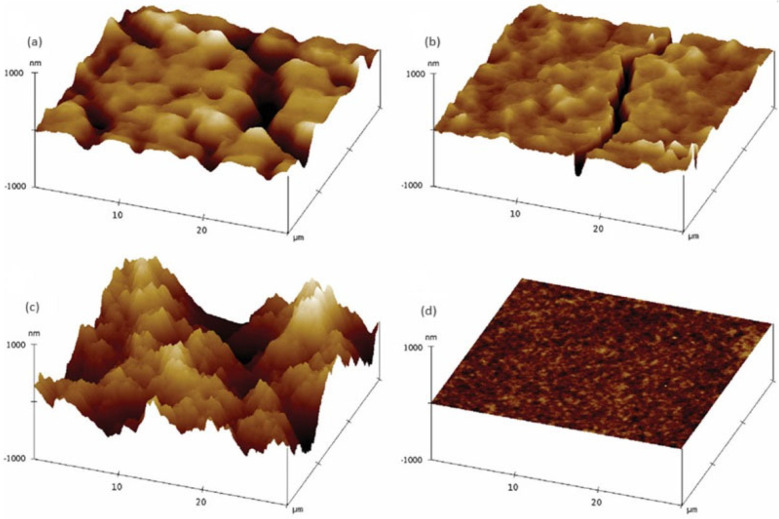
AFM morphology of (**a**) films made from original (oleic-acid-capped nanoparticles), (**b**) films made by spin-casting original nanoparticles, subsequently treated with butylamine, (**c**) films made by spin-casting nanoparticles previously exchanged to butylamine in the solution-phase, and (**d**) films made by spin-casting nanoparticles previously exchanged to ethanethiol in the solution-phase) [198] (Copyright Wiley).

The morphology of nanoparticle-based films was investigated by varying the length of ligands of the nanoparticles. Films made from oleic acid-capped PbS nanoparticles before ligand exchange showed smooth and uniform surfaces [198]. The nanoparticle-based films were treated with butylamine. The films exhibited a slight increase in surface roughness due to partial ligand exchange. In contrast, films produced from butylamine-capped PbS nanoparticles prepared by ligand exchange prior to spin coating showed a denser structure. The shorter butylamine ligands reduced the interparticle distance, creating a denser film. However, the films made by butylamine-capped PbS nanoparticles exhibited higher roughness compared to the oleic acid-capped nanoparticle-based films (Figure 5). Ethanethiol-exchanged PbS nanoparticles formed the densest and roughest films. The short ethanethiol ligands promoted closer nanoparticle packing. While the reduced interparticle distance enhances nanoparticle interaction, it also leads to greater surface roughness and irregularities, which are caused by the low dispersity of the ethanethiol-capped nanoparticles in solution. The experimental results demonstrate that shorter ligands, such as ethanethiol, produce denser but rougher films, while longer ligands like oleic acid result in smoother films, though with some voids. This highlights that the ligand length plays a crucial role in determining the final morphology of the nanoparticle-based films [198].

The morphology of films can vary significantly depending on the type of ligand used in the preparation process. For instance, in the case of TiO_2_-based films prepared with acetylacetone, a relatively smooth and uniform surface morphology is achieved. This is in contrast to films prepared with dibenzoylmethane, which result in a much rougher surface due to the nature of the dibenzoylmethane ligand, leading to a root mean square roughness as high as 52 nm. This high roughness is likely due to the hydrophobic character of dibenzoylmethane, which promotes the formation of large secondary particles during the film formation. Such variations in surface morphology, driven by the choice of ligand, play a key role in determining the properties of films, including optical transparency, electrical conductivity, and hydrophilicity [199].

### 3.3. Morphologies of Films from Nanoparticles Passivated with Inorganic Ligands

Ligand exchange from long-chain ligands of nanoparticles with inorganic ligands offers advantages in improving connectivity between particles and reducing impurities in nanoparticle-based films. The short particle-to-particle distance allows fast charge transport through the film. By incorporating an inorganic ligand, the distance between particles is reduced, imparting high electrical properties. In addition, continuous films composed of inorganic nanoparticles and matrix can be obtained after annealing the inorganic ligand-capped nanoparticle-based films.

The morphology of silver nanoparticle thin films depends on the type of halide ligand (Cl^−^, Br^−^, I^−^) used during ligand exchange. The different morphologies are primarily due to differences in the extent of sintering among the halide-functionalized nanoparticles. The interaction strength between silver and halide ligands follows the order of Ag–Cl < Ag–Br < Ag–I, based on the soft-hard acid-base theory, which classifies Ag^+^ as a soft cation and the halides (Cl^−^, Br^−^, I^−^) as soft anions with increasing softness. In Tetrabutylammonium chloride-treated silver nanoparticle films, the weakest Ag-Cl interactions resulted in slow and minimal sintering, leading to a smooth and uniform film with fewer pores and cracks. This slow sintering prevented significant nanoparticle growth, preserving the structural integrity of the films. In the case of tetrabutylammonium bromide-treated silver nanoparticle-based films, stronger Ag-Br bonds caused an intermediate level of sintering. As a result, this film exhibited more pores and cracks as the silver nanoparticles grew larger, disrupting the uniformity of the films. For tetra-n-butylammonium iodide-treated silver nanoparticle-based films, the strongest Ag-I bonds led to rapid and extensive sintering, resulting in the formation of large silver grains. This rapid growth caused significant porosity and cracking in the film as the nanoparticles coalesced and left behind empty spaces. Thus, the degree of sintering, driven by the bond strength between silver and the halide ligands, directly influenced the film morphology, with Cl^−^ yielding the smoothest films and I^−^ leading to the most porous and cracked films [163].

**Figure 6:**
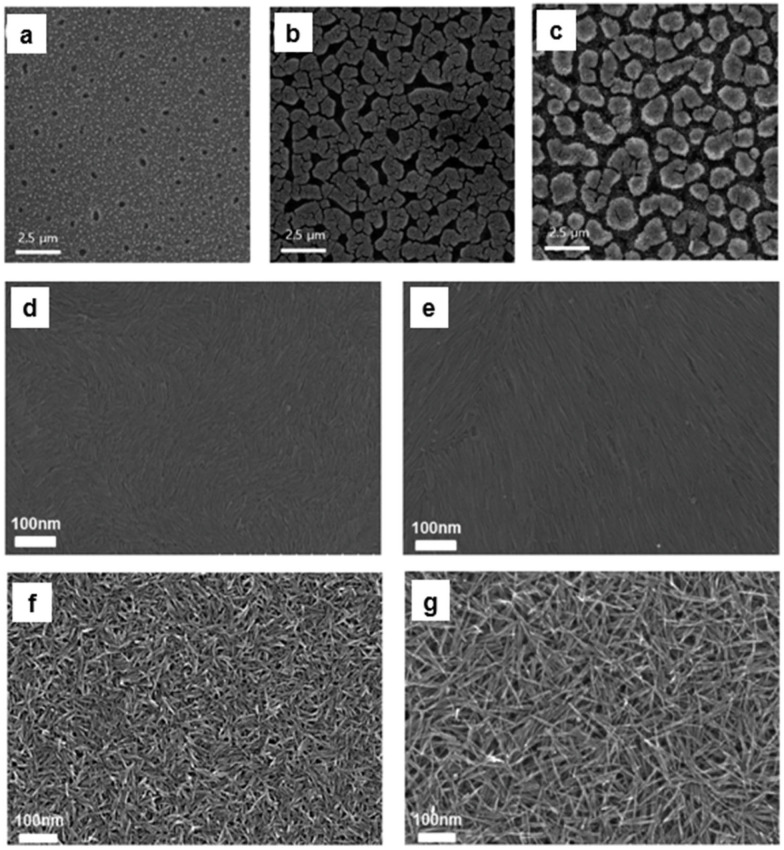
(**a**–**c**) SEM images of silver nanopartcle-based films treated for 60 s with (**a**) TBAC, (**b**) TBAB, and (**c**) TBAI (reproduced with permission [163] Copyright American Chemical Society). SEM images of (**d**,**e**) ligand-capped and (**f**,**g**) ligand-stripped low and high aspect ratio nanorod films. (Reproduced with permission [45] Copyright American Chemical Society).

The high charge density of the inorganic ligand-capped anisotropic nanorods enables the creation of porous films via strong electrostatic repulsive force, which are not obtained from the long-chain organic ligand-capped nanorods. Whereas deposition of oleylamine-capped tungsten oxide (WO_2.72_) nanorods resulted in compact packing due to the hydrophobic interaction between the long chain ligands, deposition of the nanorods after ligand-stripping using Meerwein’s salt (Et_3_OBF_4_) produced porous films due to the presence of electrostatic repulsion (Figure 6d–g). The result was the formation of a uniform mesoporous structure with high porosity without requiring additional templating agents. By adjusting the aspect ratio of the nanorods, the porosity was further tuned (Figure 6d–g). Functional electrochromic WO_x_-NbO_x_ composite films were fabricated by in-filling the pores of the nanorod-based films with niobium POM clusters on flexible substrates. The films selectively modulated visible and near-infrared light without requiring high-temperature processing. This low-temperature, template-free method is suitable for large-scale, low-cost manufacturing of flexible devices [45].

**Figure 7:**
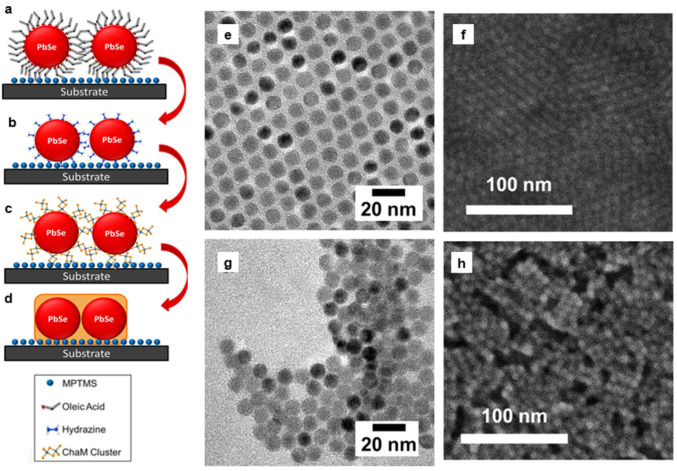
(**a**–**d**) Schematics of (**a**) a film of oleic acid-capped PbSe nanoparticles, (**b**) the film soaked in hydrazine, (**c**) the film after ligand exchange with MCC, and (**d**) the films after thermal annealing to form an all-inorganic nanocomposite. (**e**) Transmission electron microscopy (TEM) image of PbSe nanoparticles capped with oleic acid; (**f**) SEM image of the PbSe nanoparticle-based film; (**g**) TEM and (**h**) SEM images of the PbSe nanoparticles after exchanging the ligands with MCC. (Reproduced with permission [182] Copyright American Chemical Society).

Ligand exchange strategy still left gaps and smaller insulating barriers. The continuous films can be obtained from the annealing of MCC-capped nanoparticle-based films. (Figure 7) Colloidal PbSe nanoparticles, by utilizing MCC as surface ligands, are made into inorganic nanoparticle-based thin films in an inorganic matrix. The nanoparticles were immobilized on a substrate through a self-assembled monolayer, followed by a chemical treatment to replace the insulating oleate ligands with MCC clusters. Quantum confinement in PbSe nanoparticles was maintained, as indicated by the retention of excitonic features in the absorption spectrum. The PbSe nanoparticle-MCC composites exhibited high electron mobilities of 1.28 and 0.475 cm^2^ V^−^^1^ s^−^^1^ for various composite compositions [200].

### 3.4. Morphologies of Films from Nanoparticles Passivated with Polymeric Ligands

Block copolymers are sometimes intentionally used to create pores that can either be utilized directly or filled with other materials for specific applications. When block copolymer polyisoprene-b-poly(2-dimethylamino)ethyl methacrylate (PI-b-PDMAEMA) was introduced to ligand-stabilized Pt nanoparticles in a solvent mixture, the resulting film morphology became porous due to phase separation between the hydrophilic and hydrophobic blocks of the copolymer. In this system, the hydrophilic PDMAEMA block, which is originally the minority component of the block copolymer, swelled selectively upon interaction with the Pt nanoparticles, causing it to become the dominant phase in the hybrid material. This selective swelling was driven by the compatibility between the PDMAEMA block and the ligand-stabilized Pt nanoparticles, resulting in hydrophilic domains rich in nanoparticles, while the hydrophobic polyisoprene (PI) domains remained nanoparticle-free. After fabrication, the organic components of the film are removed via plasma cleaning, creating a porous structure where Pt nanoparticles remain embedded in the walls of the porous framework. The removal of organics without significant nanoparticle growth preserves the nanoscale porosity, as the nanoparticles form a rigid, stabilized network within the film. This nanoporous structure is desirable for applications requiring high surface areas, such as catalysis, because it maximizes the exposure of the nanoparticles while maintaining structural integrity. The porosity arises from the spatial separation of the block copolymer components and the selective incorporation of nanoparticles into the hydrophilic domain, which becomes the major phase due to swelling [201].

**Figure 8:**
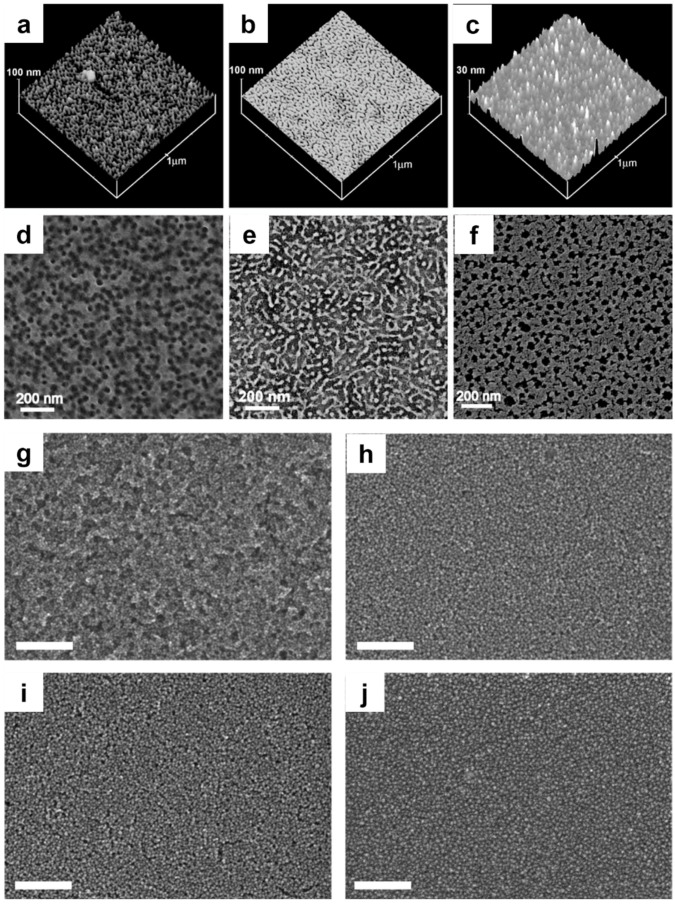
AFM height images of films with (**a**) high loading of platinum nanoparticles, (**b**) low loading of platinum nanoparticles, and (**c**) high loading of platinum nanoparticles. SEM images of (**d**) a film from high loading of platinum nanoparticles, (**e**) plasma-cleaned film with low loading of platinum nanoparticles, and (**f**) an as-made film with high loading of palladium nanoparticles (reproduced with permission [201] Copyright Wiley). SEM image of ITO nanocrystal films spray coated onto silicon substrates after PAA-mPEO_4_ wrapping in: (**g**) pH 9.1 (10% polymer by mass), (**h**) pH 8.5 (18% polymer by mass), (**i**) pH 7.9 (22% polymer by mass), and (**j**) pH 6.5 (28% polymer by mass). (Reproduced with permission [202] Copyright American Chemical Society).

The use of a polymer ligand such as PAA-mPEO4 (poly(acrylic acid)-poly(ethylene oxide)) for wrapping around nanoparticles results in stabilizing the colloidal dispersion, which leads to smoother and more uniform film morphology. Regulating the pH 9.1 to 6.5, the concentration of polymer wrapping of PAA-mPEO4 increases 10% to 28%, as does the decrease of roughness measured by AFM by 2.77 nm to 0.65 nm. The adsorption of the polymer PAA-mPEO4 onto the ITO surface is influenced by pH due to competition between the polymer and other anions for binding sites. At lower pHs (6.5), polymer adsorption is more effective, leading to higher mass retention. In contrast, at higher pH (9.1), hydroxide ions compete with the polymer for surface binding, reducing adsorption (Figure 8). The resulting films were smooth and of optical quality, whereas films made with aggregated colloids showed rough layers with noticeable porosity. This highlights the importance of polymer content in achieving the desired film uniformity and optical properties [202].

## 4. Conclusions and Outlook

The ligand exchange process plays a crucial role in determining the morphology and properties of nanoparticle-based films. By varying the types of ligands, whether they are organic, inorganic, or polymeric, it is possible to finely tune the interparticle distance, surface roughness, and overall film structure. Long-chain surfactants such as oleic acid and oleylamine provide good particle dispersion and smooth films, but they can reduce the conductivity due to the insulating nature of these ligands. On the other hand, short-chain organic ligands decrease the particle-to-particle distance, creating denser films, but they often lead to increased surface roughness and reduced film uniformity. Inorganic ligands, such as halides, enable close packing of nanoparticles, resulting in films with enhanced electrical properties. Polymeric ligands provide unique opportunities to create porous films or stabilize nanoparticles for smoother morphologies. As each ligand type brings distinct advantages, exchange of appropriate ligands prior to film deposition is a prerequisite for the desired application. This review highlights the importance of ligand selection in nanoparticle film fabrication and underscores the need for continued investigation into how different ligand systems influence the overall film morphology and properties.

As the field of nanoparticle research continues to develop, several key areas warrant further exploration to maximize the potential of nanoparticle-based films. First, there is a growing need to develop more effective ligand exchange strategies that minimize unwanted side effects such as film cracking, void formation, or the retention of insulating layers. Future efforts could focus on the use of multifunctional ligands that offer both stability and enhanced conductivity or strategies that combine different ligand types for optimized film performance. In addition, the integration of nanoparticle-based films into practical applications, such as energy storage, sensing, and flexible electronics, will require scalable, cost-effective fabrication methods. The development of green chemistry approaches for ligand exchange and film deposition will not only improve the sustainability of the process but also broaden the range of potential applications.
